# Supplementary material for: Integrated Transcriptome and Metabolome Dissecting Interaction between Vitis vinifera L. and Grapevine Fabavirus
Source: Int J Mol Sci. 2023 Feb 7;24(4):3247. doi: 10.3390/ijms24043247 (PMC9961852; doi:10.3390/ijms24043247)
Supplement: Supplementary file 1 [file ijms-24-03247-s001.zip › Figure S2.pdf]

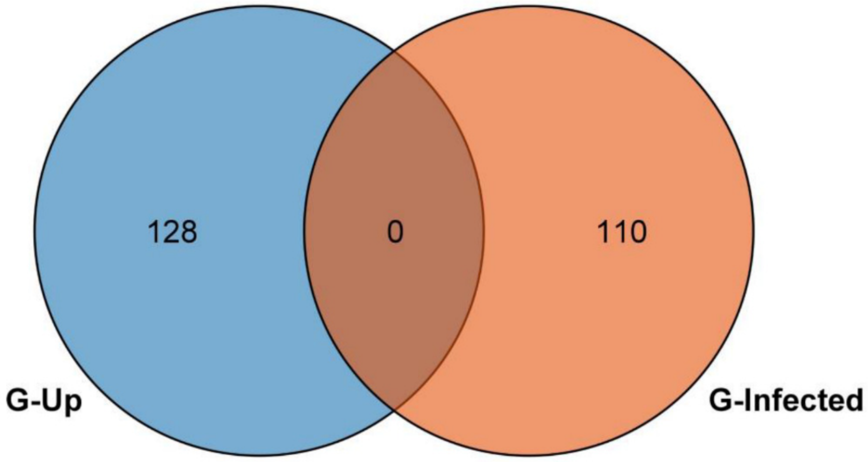

Figure S2: Venn diagram analysis of differentially expressed genes in berries. Venn diagram represents the number of up- and down-regulated genes in berries.
