# Supplementary material for: Integrated Transcriptome and Metabolome Dissecting Interaction between Vitis vinifera L. and Grapevine Fabavirus
Source: Int J Mol Sci. 2023 Feb 7;24(4):3247. doi: 10.3390/ijms24043247 (PMC9961852; doi:10.3390/ijms24043247)
Supplement: Supplementary file 1 [file ijms-24-03247-s001.zip › Figure S4.pdf]

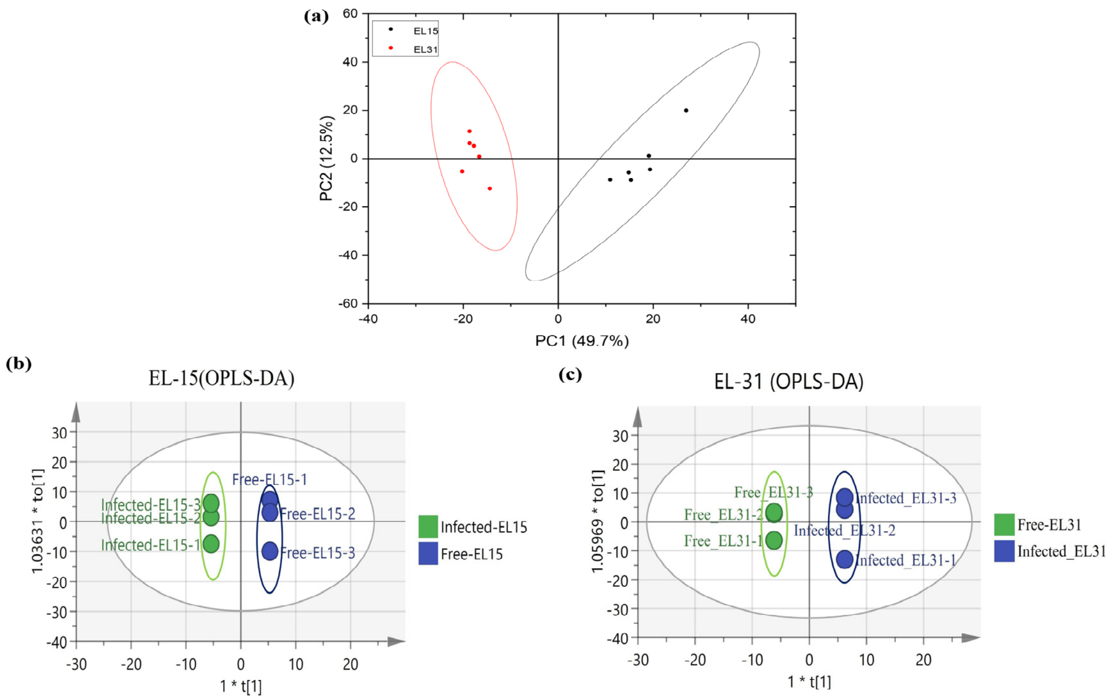

Figure S4: Principal component analysis (PCA) and orthogonal partial least squares-discriminant analysis (OPLS-DA) analyses of differentially expressed metabolites in leaves. (**a**) PCA analysis of metabolites between EL15 and EL31; (**b**) OPLS-DA score plot of DEMs between GFabV-infected and free leaves at EL15. R^2X=0.605, R^2Y=0.994, Q^2Y=0.583; (**c**) OPLS-DA score plot of DEMs at EL31. R^2X=0.415, R^2Y=0.999, Q^2Y=0.652.
