# Supplementary material for: Integrated Transcriptome and Metabolome Dissecting Interaction between Vitis vinifera L. and Grapevine Fabavirus
Source: Int J Mol Sci. 2023 Feb 7;24(4):3247. doi: 10.3390/ijms24043247 (PMC9961852; doi:10.3390/ijms24043247)
Supplement: Supplementary file 1 [file ijms-24-03247-s001.zip › Figure S5.pdf]

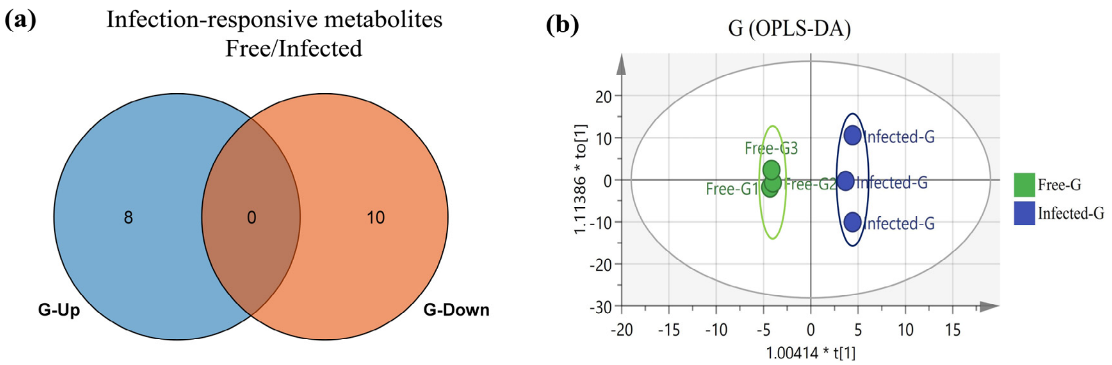


**Figure S5:** Venn diagram and orthogonal partial least squares-discriminant analysis (OPLS-DA) analyses of differentially expressed metabolites in berries. (**a**) Venn diagram of Infection-responsive differentially expressed metabolites; (**b**) OPLS-DA score plot of DEMs between GFabV-infected and GFabV-free berries. $R^2X=0.812$, $R^2Y=0.998$, $Q^2Y=0.659$.
