# Supplementary material for: Integrated Transcriptome and Metabolome Dissecting Interaction between Vitis vinifera L. and Grapevine Fabavirus
Source: Int J Mol Sci. 2023 Feb 7;24(4):3247. doi: 10.3390/ijms24043247 (PMC9961852; doi:10.3390/ijms24043247)
Supplement: Supplementary file 1 [file ijms-24-03247-s001.zip › Figure S7.pdf]

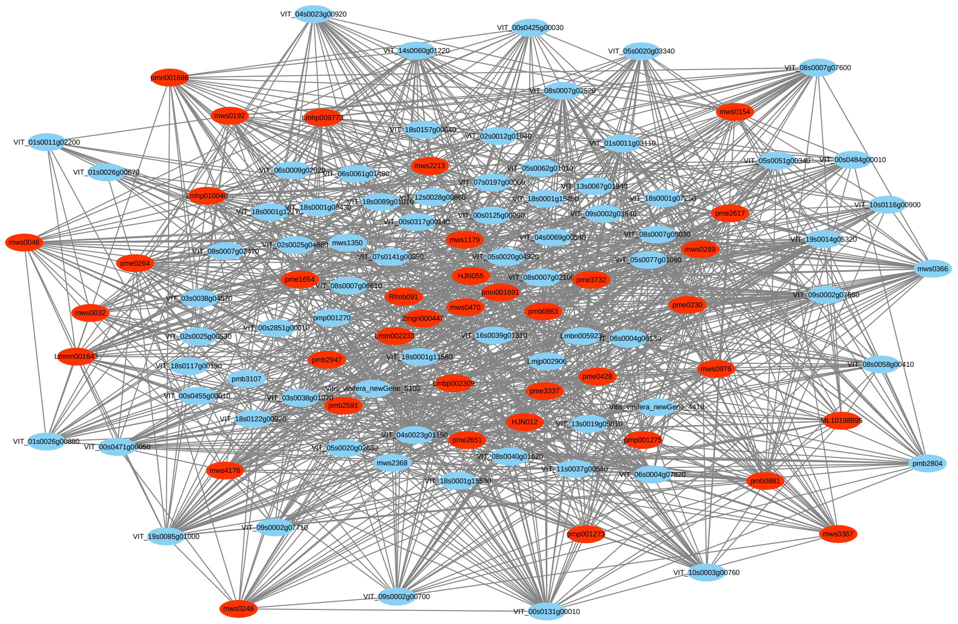

Figure S7: Analysis of transcriptome-metabolome data between GFabV-infected and GFabV-free in leaves at the EL31 stage. A coexpression network between DEGs and DEMs at EL31. Red represents the differentially expressed metabolites, blue represents the differentially expressed genes. The data were screened by the threshold: |correlation coefficient| > 0.8.
