# Supplementary material for: Integrated Transcriptome and Metabolome Dissecting Interaction between Vitis vinifera L. and Grapevine Fabavirus
Source: Int J Mol Sci. 2023 Feb 7;24(4):3247. doi: 10.3390/ijms24043247 (PMC9961852; doi:10.3390/ijms24043247)
Supplement: Supplementary file 1 [file ijms-24-03247-s001.zip › Figure S9.pdf]

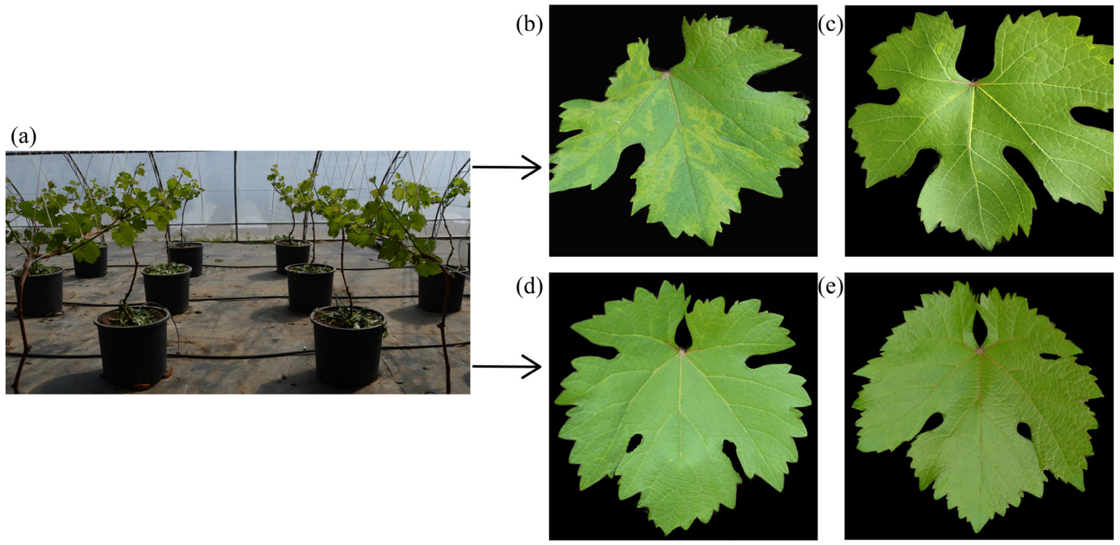

Figure S9: Physiological phenotype of leaves infected with grapevine fabavirus in ‘Summer Black’ grapevines. (a) potted grapevine samples, and Grapevine leaves: (b) infected with GFabV at EL15; (c) infected with GFabV at EL31; (d) free with GFabV at EL15; (e) free with GFabV at EL31.
