# Supplementary material for: Integrated Transcriptome and Metabolome Dissecting Interaction between Vitis vinifera L. and Grapevine Fabavirus
Source: Int J Mol Sci. 2023 Feb 7;24(4):3247. doi: 10.3390/ijms24043247 (PMC9961852; doi:10.3390/ijms24043247)
Supplement: Supplementary file 1 [file Figure S3.pdf › Figure S3.pdf]

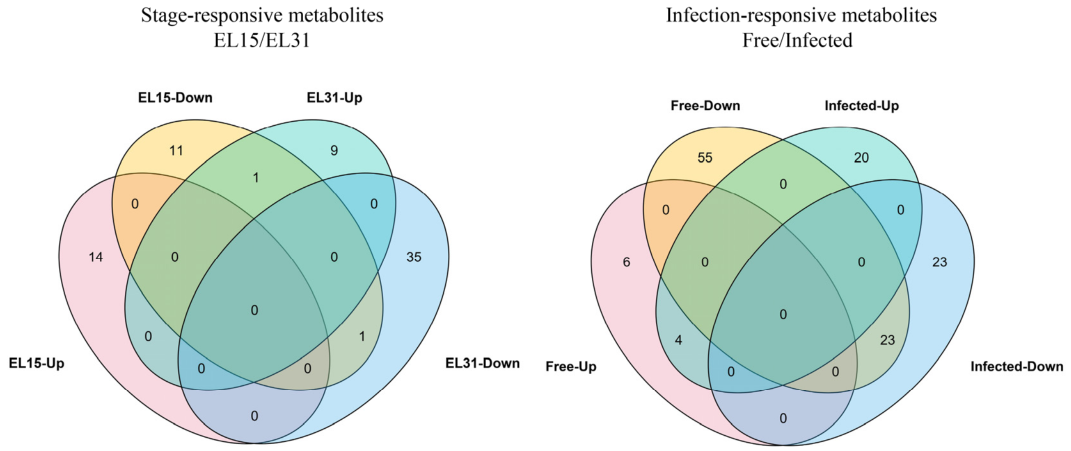

Figure S3: Venn diagrams analysis of differentially expressed metabolites in leaves. Venn diagrams different responsive matabolites between GFabV-free and GFabV-infected leaves at different stages in 2019.
